# The Mechanism of Sevoflurane Preconditioning-Induced Protections against Small Intestinal Ischemia Reperfusion Injury Is Independent of Mast Cell in Rats

**DOI:** 10.1155/2013/378703

**Published:** 2013-12-04

**Authors:** Xiaoliang Gan, Guangjie Su, Weicheng Zhao, Pinjie Huang, Gangjian Luo, Ziqing Hei

**Affiliations:** ^1^Department of Anesthesiology, The Third Affiliated Hospital, Sun Yat-sen University, Guangzhou 510630, China; ^2^Department of Anesthesiology, Zhongshan Ophthalmic Center, Sun Yat-sen University, Guangzhou 510060, China; ^3^Department of Anesthesiology, The First People's Hospital of Foshan, Foshan 528000, China

## Abstract

The study aimed to investigate whether sevoflurane preconditioning can protect against small intestinal ischemia reperfusion (IIR) injury and to explore whether mast cell (MC) is involved in the protections provided by sevoflurane preconditioning. Sprague-Dawley rats exposed to sevoflurane or treated with MC stabilizer cromolyn sodium (CS) were subjected to 75-minute superior mesenteric artery occlusion followed by 2-hour reperfusion in the presence or absence of MC degranulator compound 48/80 (CP). Small intestinal ischemia reperfusion resulted in severe intestinal injury as demonstrated by significant elevations in intestinal injury scores and p47^phox^ and gp91^phox^, ICAM-1 protein expressions and malondialdehyde and IL-6 contents, and MPO activities as well as significant reductions in SOD activities, accompanied with concomitant increases in mast cell degranulation evidenced by significant increases in MC counts, tryptase expression, and **β**-hexosaminidase concentrations, and those alterations were further upregulated in the presence of CP. Sevoflurane preconditioning dramatically attenuated the previous IIR-induced alterations except MC counts, tryptase, and **β**-hexosaminidase which were significantly reduced by CS treatment. Furthermore, CP exacerbated IIR injury was abrogated by CS but not by sevoflurane preconditioning. The data collectively indicate that sevoflurane preconditioning confers protections against IIR injury, and MC is not involved in the protective process.

## 1. Introduction

Small intestinal ischemia reperfusion (IIR) injury occurs frequently in many clinical conditions, including bowel transplantation [1] and liver transplantation, as well as all kinds of shock [2]. Although the advanced treatments have been applied in clinical, the mortality associated with IIR is still high [3]. Mast cells are widely present throughout gastrointestinal tract; previous studies including ours have demonstrated that mast cells play a critical role in the pathogenesis of IIR injury [4, 5], and mast cell inhibition provides a promising therapeutic method against IIR injury.

Sevoflurane, a novel inhaled anesthetic, has been widely used in patients undergoing surgery. In addition to its anesthesia effect, several studies so far have demonstrated that sevoflurane preconditioning confers protections against hypoxic and ischemic cerebral and spinal cord injuries [6, 7], moreover, sevoflurane preconditioning also provides promising benefits against ischemia/reperfusion injury in the heart and kidneys [8, 9]. The protective mechanisms were associated with the reduction of leukocytes infiltration [10], downregulation of apoptosis [6], and enhancement of antioxidant enzymes [11]. However, to our knowledge, there is no evidence supporting the role of sevoflurane preconditioning in IIR injury, and the underlying mechanism is incompletely understood.

Several studies so far have demonstrated that sevoflurane can be safely used in patients diagnosed to have mastocytosis (a group of rare disorders caused by the presence of too many mast cells) without triggering mast cell degranulation with release of histamine, prostaglandin, tryptase, and heparin [12]. By contrast, Annecke reported that sevoflurane preconditioning can attenuate heart ischemia reperfusion injury without inhibiting mast cell release histamine [13]. However, the direct relationship between sevoflurane preconditioning and mast cell degranulation remains to be elucidated.

Therein, we aimed to investigate whether sevoflurane preconditioning can provide protections against IIR injury; in particular, we studied whether mast cell was involved in the protections provided by sevoflurane preconditioning by using a specific mast cell degranulator (Compound 48/80) and a specific stabilizer (cromolyn sodium) in a rodent model.

## 2. Materials and Methods

### 2.1. Animal Experiments

Female Sprague-Dawley (SD) rats weighing 180–200 g, purchased from the Animal Center of Guangdong Province (Guangzhou, China), were housed individually in wire-bottomed cages and were placed under pathogen free condition for one week before use. The experimental protocol and design were approved by the Sun Yat-sen University Animal Experimentation Committee and performed according to Sun Yat-sen University Guidelines for Animal Experimentation. All the animals were allowed free access to water and food ad libitum except 16 h before surgery. Rats were randomly divided into seven groups: (1) Sham-operated (SH), (2) sole IIR (IIR), (3) IIR + Compound 48/80 (IIR + CP), (4) IIR + cromolyn sodium (IIR + CS), (5) IIR + cromolyn sodium + Compound 48/80 (IIR + CS + CP), (6) sevoflurane + IIR (SEV + IIR), and (7) sevoflurane + IIR + Compound 48/80 (SEV + IIR + CP). In sevoflurane pretreated groups, the rats were exposed to rats 2.3% sevoflurane in a gas-tight anesthesia chamber for 1 hour according to previous studies for 3 subsequent days [7]; the other rats were exposed to oxygen alone. At the 4th day, the rats were anesthetized by intraperitoneal injection of 10% of chloral hydrate (3.5 mL/kg) after fasting for 16 h, and the abdomen was opened by a midline incision in a supine position; the superior mesenteric artery (SMA) was isolated and occluded for 75 min with a small clamp, and then the clamp was released to maintain the rats for 2 h during reperfusion in all IIR groups. However, in the Sham-operated group, the SMA was just isolated but not clamped and maintained the same period during the surgery. The cromolyn sodium (mast cell stabilizer, 50 mg/kg) was injected intravenously at 15 min after ischemia, and the Compound 48/80 (mast cell degranulator, 0.75 mg/kg) was intravenously injected via the tail vein at 5 min before the release of the clamp in the Compound 48/80 groups. Meanwhile, the same volumes of physiological saline were administrated in the control groups. The doses of agents were adjusted in accordance with our previous study [5]. During the surgery, all the rats body temperature was maintained at 38°C using heated pad. And 10 mL/kg 37°C normal saline was injected subcutaneously to avoid dehydration after the abdomen had been closed.

### 2.2. Collection of Intestinal Mucosa

At the end of the experiment, the whole small intestine was removed carefully, and a segment of 1.0 cm intestine was cut from 10 cm to terminal ileum and fixed in 10% formaldehyde and then embedded in paraffin for section. The remaining small intestine was washed thoroughly with 0°C normal saline and then opened longitudinally to expose the intestinal epithelium, after being rinsed completely with 0°C normal saline and dried with suction paper. The mucosal layer was harvested by gentle scraping of the epithelium with a glass slide with a plate on the ice and then was stored at −70°C for further measurements.

### 2.3. Intestinal Histology

Five-*μ*m thick sections were prepared from paraffin-embedded intestine tissue, the segment of small intestine was stained with hematoxylin-eosin. And the damages of intestinal mucosa were evaluated by two histologists who were initially blinded to the experiment according to the Chiu's standard [14]. Criteria of Chiu grading system consists from 5 subdivisions according to the changes of villus and gland of intestinal mucosa: grade 0, normal mucosa; grade 1, development of subepithelial Gruenhagen's space at the tip of villus; grade 2, extension of the space with moderate epithelial lifting; grade 3, massive epithelial lifting with a few denuded villi; grade 4, denuded villi with exposed capillaries; and grade 5, disintegration of the lamina propria, ulceration and hemorrhage.

### 2.4. Detection of the Content of Malondialdehyde (MDA) in Small Intestinal Mucosa

Small intestinal mucosa was homogenized with normal saline. The tissue content of MDA, an index of oxidative stress mediated tissue lipid peroxidation, was determined by the TBA method (Jiancheng Bioengineering Ltd, Nanjing, China) as described in [15]. The values of MDA in tissue homogenate were expressed as nmol/mL. The content of final MDA in small intestinal mucosa was normalized for tissue weight.

### 2.5. Detection of Superoxide Dismutase (SOD) Activity in Small Intestinal Mucosa

Small intestinal mucosa was made into a homogenate with normal saline, frozen at −20°C for 5 min and centrifuged for 15 min at 4000 r/min. Supernatants were transferred into fresh tubes for the evaluation of SOD activity. SOD activity was assessed by SOD detection kits according to the manufacturer's instructions (Jiancheng Bioengineering Ltd, Nanjing, China). Presented data were normalized for tissue weight.

### 2.6. Measurement of *β*-Hexosaminidase Level in Serum

At the end of the experiment, 2 mL of blood was obtained from inferior vena cava and was centrifuged for 15 min at 4000 r/min. The supernatant, serum, was stored at −20°C for the determination of *β*-hexosaminidase level using the modification of a previously described method [16]. Briefly, 50 *μ*L of serum was incubated with 50 *μ*L of 1 mM p-nitrophenyl-N-acetyl-*β*-D-glucosaminide dissolved in 0.1 M citrate buffer (pH 5) in 96-well plate at 37°C for 1 h. The reaction was terminated with 200 *μ*L/well of 0.1 M carbonate buffer (pH 10.5). The absorbance at 405 nm was measured using a microplate reader.

### 2.7. Western Blotting

Intestinal mucosa samples were homogenized with lysis buffer for 30 seconds in a mortar and pestle with liquid nitrogen. Homogenates were centrifuged at 13000 rpm for 10 min at 4°C and the supernatant was collected as the source of sample of protein. The proteins were processed with standard methods for western blot analysis as described in [17]. Rat monoclonal antitryptase antibody, rat monoclonal anti-gp91^phox^ and anti-p47^phox^ antibodies, and rat monoclonal anti-ICAM-1 and *α*-tubulin antibodies were obtained from Santa Cruz (Santa Cruz, CA, USA). The secondary antibody conjugated to horseradish peroxidase was diluted at 1 : 2,000 (Santa Cruz, CA, USA). Immunoblots were incubated with an enhanced chemiluminescence detection system (KeyGen Biotech, China) and the densitometry analysis was performed using Quantity One software.

### 2.8. Determination of IL-6 Production in Small Intestinal Mucosa by Enzyme Immunoassay

Briefly, intestinal protein was measured by BCA Protein Assay Kit provided by KenGen Biotech Company, Nanjing, China; the results were expressed as g/L. And the levels of IL-6 were measured by commercial ELISA kits following manufacturer's instructions (R&D systems Inc., USA). The absorbance was read at 450 nm by a biokinetics microplate reader Model EL340 (Biotek Instruments, USA); the results were expressed as pg/L; then the levels of IL-6 in the intestine were calculated as pg/mg protein.

### 2.9. Determination of Myeloperoxidase (MPO) Activity in Small Intestinal Mucosa

Myeloperoxidase (MPO) activity was determined with the O-dianisidine method [18], using a MPO detection kit (Nanjing Jiancheng Bioengineering Institute) as we described [19]. MPO activity was defined as the quantity of enzyme degrading 1 *μ*mol of peroxide per minute at 37°C and was expressed in units per gram weight of wet tissue.

### 2.10. Assessment of Mast Cell Counts in Small Intestine

Five *μ*m thick sections were prepared from paraffin-embedded intestine tissue; after deparaffinization, endogenous peroxidase was quenched with 3% H_2_O_2_ in deionized water for 10 min. Nonspecific binding sites were blocked by incubating the sections in 10% of normal rabbit serum for 1 h. The sections were then incubated with polyclonal rat antimast cell tryptase (dilution 1 : 2000, Santa Cruz, CA, USA) for 20 min at 37°C, followed by incubation with biotinylated mouse anti-rat IgG at room temperature for 10–15 min. After 3 × 5 min PBS rinses, the horseradish-peroxidase-conjugated streptavidin solution was added and incubated at room temperature for 10–15 min. The antibody binding sites were visualized by incubation with a diaminobenzidine-H_2_O_2_ solution. The sections incubated with PBS instead of the primary antibody were used as negative controls. Brown-yellow granules in cytoplasm were recognized as positive staining for tryptase. We calculated the tryptase positive mast cells in five randomly selected areas at ×200 magnification by Image-Pro Plus 5.0 (USA).

### 2.11. Statistical Analysis

The data (except for the survival curves) were expressed as mean ± SEM. Analysis of variance was performed using Graphpad Prism software. One-way analysis of variance was used for multiple comparisons, followed by Bonferroni's and Student's *t*-test for unpaired values. The survival rate was expressed as the percentage of live animals, and the Mantel Cox log rank test was used to determine differences between survival curves. A *P* value less than 0.05 is considered a significant difference.

## 3. Results

### 3.1. Effects of Sevoflurane Preconditioning on Survival Rates in Rats Challenged to IIR in the Presence or Absence of Mast Cell Activator

**Figure 1:**
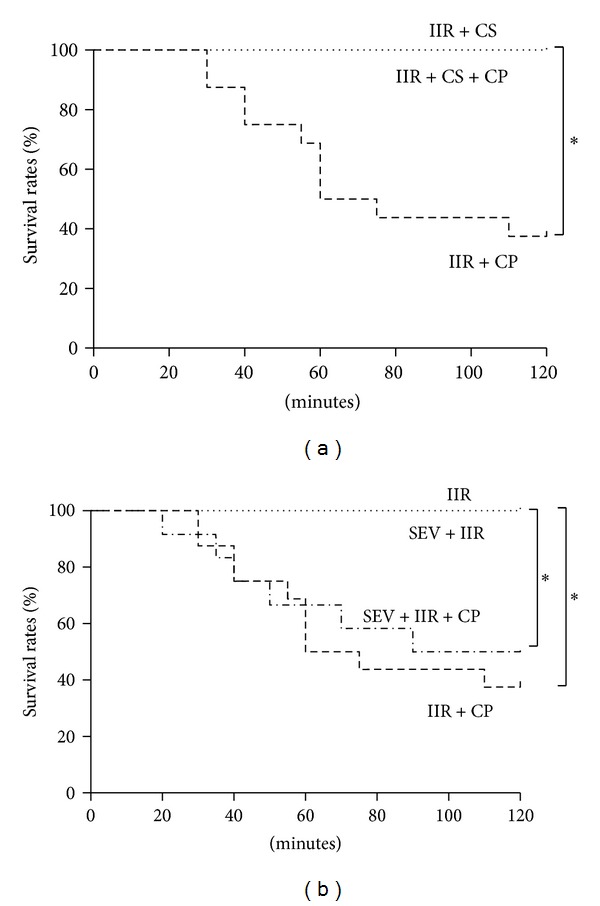
Survival rates after IIR injury. SH group (Sham-operated group), IIR group (75 min intestinal ischemia and 2 h reperfusion), IIR + CP group (IIR group + Compound 48/80 1 mg/kg), IIR + CS group (50 mg/kg cromolyn sodium treated IIR group), IIR + CS + CP group (50 mg/kg cromolyn sodium treated IIR group + Compound 48/80 1 mg/kg), SEV + IIR group (2.3% sevoflurane pretreated IIR group), SEV + IIR + CP group (2.3% sevoflurane pretreated IIR group + Compound 48/80 1 mg/kg). The survival rates in 2 h after reperfusion were 100% in all groups except IIR + CP and SEV + IIR + CP groups. Results are expressed as percentage of live animals, *n* = 6 per group, whereas *n* = 16 in IIR + CP group and *n* = 12 in SEV + IIR + CP group. *indicated that *P* value was less than 0.05.

Initially, we sought to explore the role of sevoflurane preconditioning in the IIR injury; pretreatment of rats with sevoflurane displayed no significant differences in comparison with IIR group. And we also sought to investigate whether mast cell inhibition is involved in the benefits induced by sevoflurane preconditioning; the mast cell special stabilizer and activator were addressed as positive and negative control groups. As illustrated in Figure 1, activation of mast cell by Compound 48/80 resulted in significant decreases in 2 h survival rates after the clamp releasing as compared with sole IIR group, and stabilizing mast cell dramatically abolished the reductions in 2 h survival induced by Compound 48/80; by contrast, pretreatment of sevoflurane did not attenuate Compound 48/80 mediated exacerbation of postischemic survival rate in rats subjected to IIR. These data further demonstrate that mast cell degranulation exacerbates IIR injury, and the results gave us the first impression that sevoflurane preconditioning may not contribute to mast cell stabilization.

### 3.2. Effects of Sevoflurane Preconditioning on Small Intestinal Structure in Rats Undergoing IIR in the Presence or Absence of Mast Cell Activator

**Figure 2:**
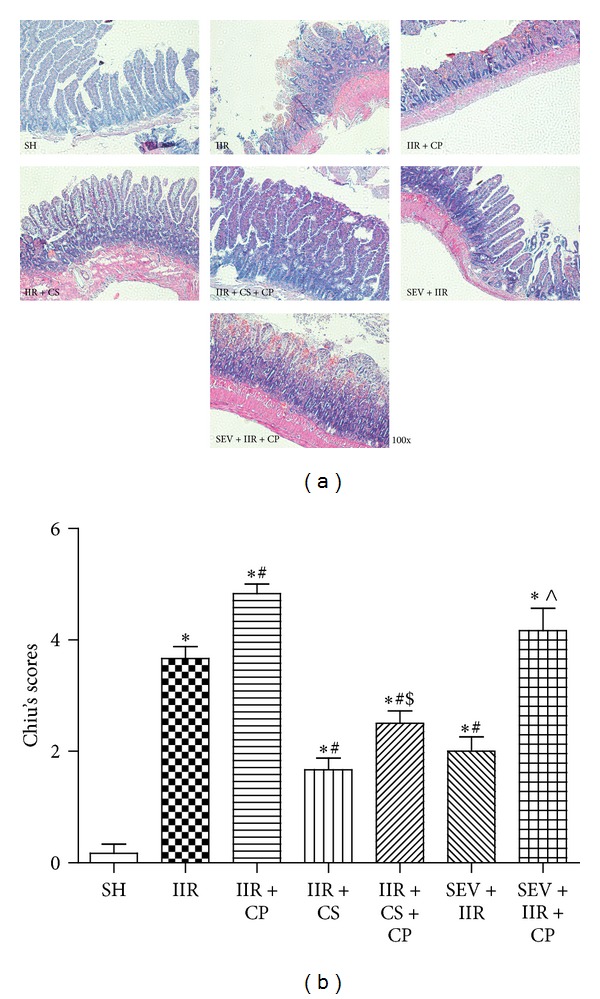
Morphological changes of intestine and intestinal histological score under light microscope after IIR injury. (a) is representative image of SH group (Sham-operated group), IIR group (75 min intestinal ischemia and 2 h reperfusion), IIR + CP group (IIR group + Compound 48/80 1 mg/kg), IIR + CS group (50 mg/kg cromolyn sodium treated IIR group), IIR + CS + CP group (50 mg/kg cromolyn sodium treated IIR group + Compound 48/80 1 mg/kg), SEV + IIR group (2.3% sevoflurane pretreated IIR group), and SEV + IIR + CP group (2.3% sevoflurane pretreated IIR group + Compound 48/80 1 mg/kg), (HE staining, ×100). Bar graph in (b) quantified the intestine histological scores. Results are expressed as Mean ± SEM. *n* = 6 per group. *P < 0.05 versus SH group, ^#^ *P* < 0.05 versus IIR group, ^$^ *P* < 0.05 versus IIR + CP group, ^&^ *P* < 0.05 versus IIR + CS group, and ^∧^ *P* < 0.05 versus SEV + IIR group.

Because there were no differences in the 2 h survival among IIR group, IIR + CS group, and SEV + IIR group, we, next, sought to define the further effects of sevoflurane preconditioning on the IIR injury and its relationships with mast cell. The sections of small intestine obtained for evaluation of the injury severity by HE staining at the end of the experiment, 75 min ischemia followed by 2 h reperfusion, resulted in severe damages to small intestine; as depicted in Figure 2, multiple erosions and bleeding were observed in IIR group, while Compound 48/80 further aggravated IIR injury demonstrated by more multiple erosions and bleedings and more inflammatory cell sequestrations seen in the IIR + CP group, whereas the villus and glands were normal and no inflammatory cell infiltration was observed in mucosal epithelial layer in Sham-operated group. Mast cell stabilizer and sevoflurane similarly significantly attenuated the injuries in small intestine and only slight edema of mucosa villus and infiltration of few necrotic epithelial inflammatory cells were found in mucosa epithelial layer under microscopy assessment in CS or sevoflurane treated groups. However, cromolyn sodium, but not sevoflurane, blocked Compound 48/80-induced exacerbation in small intestinal morphology changes after 2 h reperfusion. Consistent with morphological changes, the Chiu's scores markedly increased in IIR group as compared with Sham-operated group while treated with mast cell degranulator Compound 48/80 after ischemia resulted in further increases in Chiu's scores. cromolyn sodium and sevoflurane similarly lowered the Chiu's scores; of note, cromolyn sodium, but not sevoflurane, significantly limited the changes induced by Compound 48/80 (*P* < 0.05, IIR + CS + CP versus IIR + CP group).

### 3.3. Effects of Sevoflurane Preconditioning on Mast Cell Degranulation in Small Intestine in Rats Undergoing IIR in the Presence or Absence of Mast Cell Activator. 

**Figure 3:**
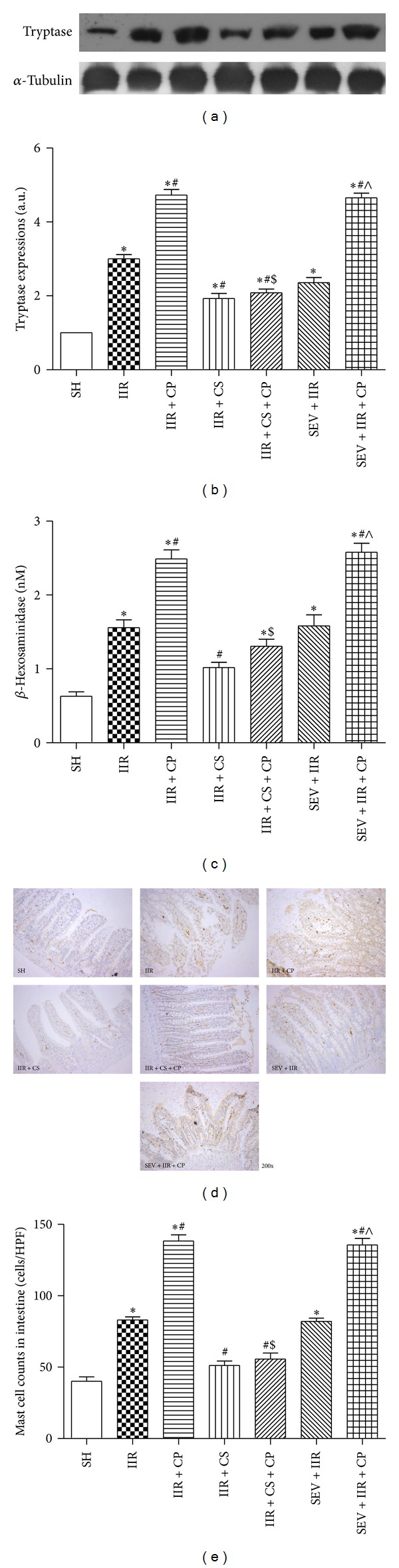
Tryptase protein expression in intestine mucosa, and *β*-hexosaminidase levels in serum, intestinal mast cell counts after IIR injury. SH group (Sham-operated group), IIR group (75 min intestinal ischemia and 2 h reperfusion), IIR + CP group (IIR group + Compound 48/80 1 mg/kg), IIR + CS group (50 mg/kg cromolyn sodium treated IIR group), IIR + CS + CP group (50 mg/kg cromolyn sodium treated IIR group + Compound 48/80 1 mg/kg), SEV + IIR group (2.3% sevoflurane pretreated IIR group), and SEV + IIR + CP group (2.3% sevoflurane pretreated IIR group + Compound 48/80 1 mg/kg). Representative band of tryptase protein expression in intestinal mucosa is displayed in (a). Bar graphs (b) quantified tryptase protein expression (*n* = 3). Bar graph (c) quantified *β*-hexosaminidase levels (*n* = 6). Representative images of immunohistochemical staining for tryptase in intestine are displayed in (d) (SP staining, ×200); brown-yellow granules in the cytoplasm were recognized as mast cells. Bar graph in (e) quantified mast cell counts in small intestine (*n* = 6). Results are expressed as Mean ± SEM. *P < 0.05 versus SH group, ^#^ *P* < 0.05 versus IIR group, ^$^ *P* < 0.05 versus IIR + CP group, ^&^ *P* < 0.05 versus IIR + CS group, and ^∧^ *P* < 0.05 versus SEV + IIR group.

Tryptase and *β*-hexosaminidase are the unique markers released from mast cell and the elevations can be recognized as mast cell degranulation. As shown in Figure 3, after 2 h of reperfusion, we found that tryptase protein expression and *β*-hexosaminidase level, as well as mast cell counts, were greatly increased in group IIR as compared with Sham-operated group, and Compound 48/80 resulted in further mast cell degranulation as significant increases in tryptase protein expression, *β*-hexosaminidase level, and mast cell counts were more observed in group IIR + CP than in group IIR; as expected, mast cell stabilizer CS not only attenuated the upregulations induced by IIR, but also abolished the exacerbations mediated by Compound 48/80. Interestingly, pretreatment of rats with sevoflurane exhibited no reductions in tryptase protein expression, *β*-hexosaminidase level, and mast cell counts induced by IIR challenge; furthermore, the alterations were further aggravated in the presence of Compound 48/80 despite sevoflurane preconditioning, which was comparable to IIR + CP group. Collectively, the findings from the current study indicated that mast cell degranulation aggravated IIR injury and mast cell stabilization is not involved in the protections provided by sevoflurane preconditioning.

### 3.4. Effects of Sevoflurane Preconditioning on Neutrophil Rolling in Small Intestine in Rats Undergoing IIR in the Presence or Absence of Mast Cell Activator

**Figure 4:**
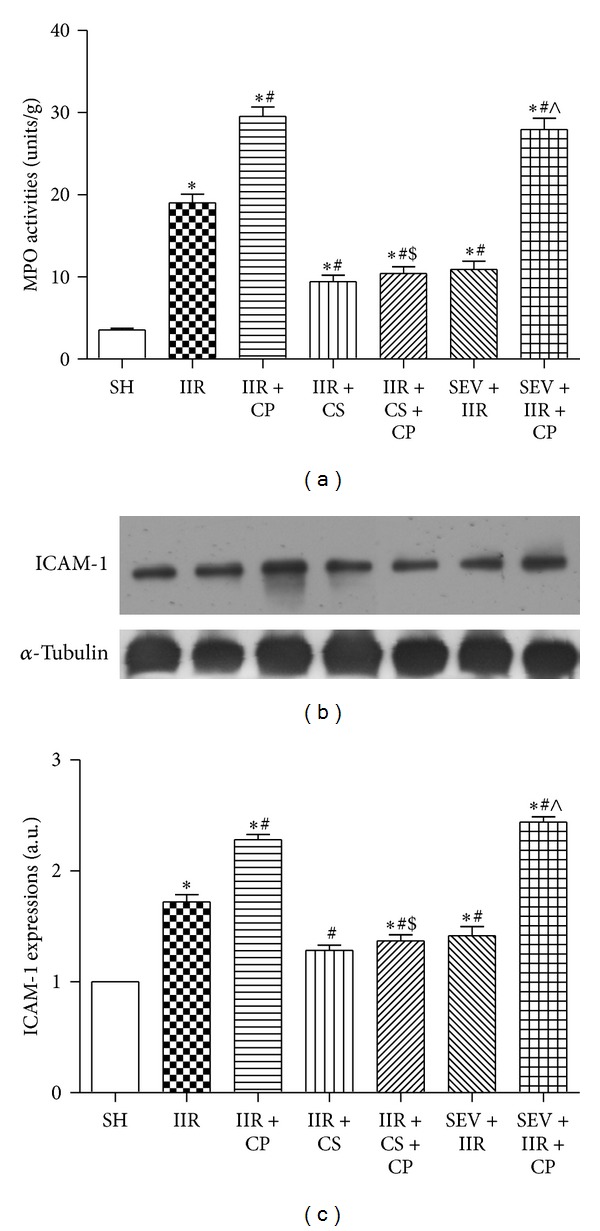
MPO activities and ICAM-1 protein expression in intestine mucosa after IIR injury. SH group (Sham-operated group), IIR group (75 min intestinal ischemia and 2 h reperfusion), IIR + CP group (IIR group + Compound 48/80 1 mg/kg), IIR + CS group (50 mg/kg cromolyn sodium treated IIR group), IIR + CS + CP group (50 mg/kg cromolyn sodium treated IIR group + Compound 48/80 1 mg/kg), SEV + IIR group (2.3% sevoflurane pretreated IIR group), and SEV + IIR + CP group (2.3% sevoflurane pretreated IIR group + Compound 48/80 1 mg/kg). Bar graph (a) quantified MPO activities in intestinal mucosa (*n* = 6). Representative band of ICAM-1 protein expressions is displayed in (b). Bar graphs (c) quantified ICAM-1 protein expressions (*n* = 3). Results are expressed as Mean ± SEM. *P < 0.05 versus SH group, ^#^ *P* < 0.05 versus IIR group, ^$^ *P* < 0.05 versus IIR + CP group, ^&^ *P* < 0.05 versus IIR + CS group, and ^∧^ *P* < 0.05 versus SEV + IIR group.

IIR injury is characterized by neutrophil infiltration into the inflamed tissues [20], as illustrated in Figure 4; in agreement with previous results [20], we also found that IIR led to marked increases in MPO activities and ICAM-1 protein expressions as compared with Sham-operated group; furthermore, Compound 48/80 led to further increases in MPO activities and ICAM-1 protein expression in group IIR + CP than in group IIR. Administrations with cromolyn sodium and sevoflurane similarly significantly inhibited neutrophil infiltration/activation demonstrated by downregulation of MPO activities and ICAM-1 protein expression induced by IIR. Of note, mast cell stabilizer, but not sevoflurane, blocked Compound 48/80 mediated exacerbation of IIR by reducing neutrophil infiltration.

### 3.5. Effects of Sevoflurane Preconditioning on Inflammation in Small Intestine in Rats Undergoing IIR in the Presence or Absence of Mast Cell Activator

**Figure 5:**
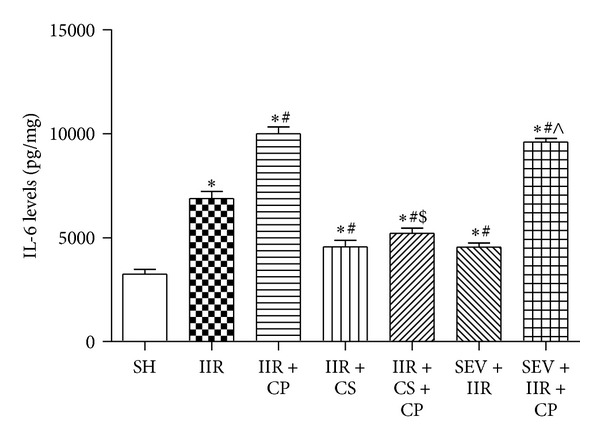
Levels of IL-6 in intestine after IIR injury. SH group (Sham-operated group), IIR group (75 min intestinal ischemia and 2 h reperfusion), IIR + CP group (IIR group + Compound 48/80 1 mg/kg), IIR + CS group (50 mg/kg cromolyn sodium treated IIR group), IIR + CS + CP group (50 mg/kg cromolyn sodium treated IIR group + Compound 48/80 1 mg/kg), SEV + IIR group (2.3% sevoflurane pretreated IIR group), and SEV + IIR + CP group (2.3% sevoflurane pretreated IIR group + Compound 48/80 1 mg/kg). Bar graph quantified IL-6 levels in intestinal mucosa. Results are expressed as Mean ± SEM. *n* = 6 per group. *P < 0.05 versus SH group, ^#^ *P* < 0.05 versus IIR group, ^$^ *P* < 0.05 versus IIR + CP group, ^&^ *P* < 0.05 versus IIR + CS group, and ^∧^ *P* < 0.05 versus SEV + IIR group.

Inflammatory cytokine, interleukin 6 (IL-6), has been demonstrated to be implicated in the pathogenesis of IIR injury [21]. As shown in Figure 5, in the current study, rats undergoing 75 min ischemia and 2 h reperfusion showed significant increases in IL-6 levels in small intestinal mucosa in group IIR as compared with Sham-operated group; moreover, administration with Compound 48/80 further resulted in dramatic enhancements in IL-6 levels in group IIR + CP (*P* < 0.05 versus group IIR). Mast cell stabilizer cromolyn sodium and sevoflurane similarly greatly abrogated the increases in IL-6 levels; however, cromolyn sodium, but not sevoflurane, blocked the enhancements in IL-6 levels resulted from IIR in the presence of the MC activator Compound 48/80.

### 3.6. Effects of Sevoflurane Preconditioning on Oxidative Stress in Small Intestine in Rats Undergoing IIR in the Presence or Absence of Mast Cell Activator

**Figure 6:**
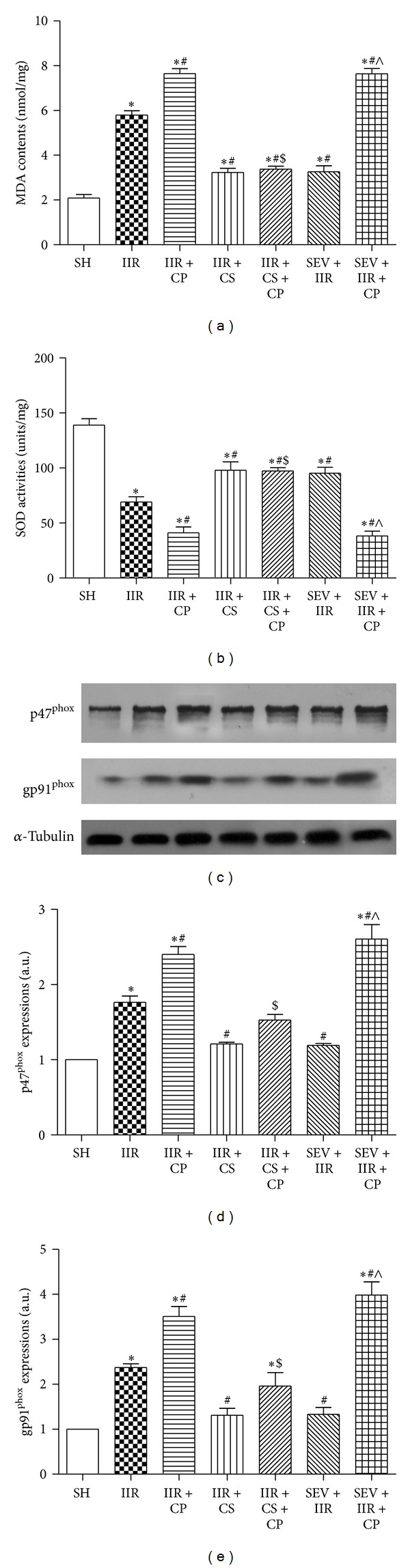
MDA contents and SOD activities and p47^phox^ and gp91^phox^ protein expressions in intestine mucosa after IIR injury. SH group (Sham-operated group), IIR group (75 min intestinal ischemia and 2 h reperfusion), IIR + CP group (IIR group + Compound 48/80 1 mg/kg), IIR + CS group (50 mg/kg cromolyn sodium treated IIR group), IIR + CS + CP group (50 mg/kg cromolyn sodium treated IIR group + Compound 48/80 1 mg/kg), SEV + IIR group (2.3% sevoflurane pretreated IIR group), and SEV + IIR + CP group (2.3% sevoflurane pretreated IIR group + Compound 48/80 1 mg/kg). Bar graph (a) quantified MDA contents and bar graph (b) quantified SOD activities, *n* = 6 per group. Representative bands of p47^phox^ and gp91^phox^ protein expressions are displayed in (c). Bar graphs (d) and (e) quantified p47^phox^ and gp91^phox^ protein expressions, respectively (*n* = 3). Results are expressed as Mean ± SEM. *P < 0.05 versus SH group, ^#^ *P* < 0.05 versus IIR group, ^$^ *P* < 0.05 versus IIR + CP group, ^&^ *P* < 0.05 versus IIR + CS group, and ^∧^ *P* < 0.05 versus SEV + IIR group.

Since mast cell inhibition is not involved in the protection induced by sevoflurane preconditioning, we sought other underlying mechanisms by which sevoflurane preconditioning provides benefits against IIR. Ischemia reperfusion injury is also characterized by up-regulation of free radical species [17]. In line with previous results [22], as shown in Figure 6, 75 min ischemia followed by 2 h reperfusion resulted in substantial increases in MDA contents and reductions in SOD activities as compared with Sham-operated group. Meanwhile, IIR also caused great increases in gp91^phox^ and p47^phox^ protein expression when compared with Sham-operated group. Moreover, Compound 48/80 further aggravated the changes of oxidative stress induced by IIR (*P* < 0.05 IIR + CP versus IIR); by contrast, cromolyn sodium and sevoflurane similarly attenuated IIR mediated oxidative stress demonstrated by downregulating MDA contents and gp91^phox^ and p47^phox^ protein expressions and up-regulating SOD activities in cromolyn sodium and sevoflurane treated groups by comparison with group IIR. Furthermore, cromolyn sodium limited the further increases of oxidative stress induced by Compound 48/80. As expected, sevoflurane preconditioning exhibited no protective effects against Compound 48/80 exacerbated oxidative stress.

## 4. Discussion

We have shown in the current study, to our knowledge, for the first time that sevoflurane preconditioning attenuated the small intestinal ischemia reperfusion injury; inhibiting neutrophil sequestration may represent the major mechanism whereby sevoflurane preconditioning alleviates IIR injury. Further, we showed that the subsequent mast cell activation contributed to the exacerbation of IIR injury demonstrated by significant increases in intestinal injury score and elevations in postischemic oxidative stress and neutrophil sequestrations, leading to significantly reduced postischemic survival; stabilizing mast cell by cromolyn sodium not only dramatically attenuated IIR injury, but also totally blocked the exacerbated injury induced by mast cell activation. By contrast, we showed that sevoflurane preconditioning offers no protections in mast cell mediated IIR injury.

Mast cell, containing a large range of mediators, presents throughout gastrointestinal tract. Although mast cell may function as host defense to prevent bacterial invasion [23], prolonged MC activation has been demonstrated to contribute to the development of a variety of disorders and to play a critical role in the pathogenesis of IIR injury by releasing histamine, tryptase, and TNF-*α* [24]. Ramos et al. reported that mast cell degranulation plays a significant role in the development of sepsis by regulating cell death, which resulted in multiorgan dysfunction [25]. Consistent with the previous findings that stabilizing MC from degranulation would be one of the potential strategies for combating IIR injury [26], our current study further revealed that inhibiting mast cell from activation/degranulation by cromolyn sodium significantly limited the IIR injury evidenced as increases in postischemic survival and reductions in tryptase protein expression and *β*-hexosaminidase levels, which causes or exacerbates IIR injury; this notion is further supported by the fact that mast cell stabilizer cromolyn sodium abolished the exacerbated injury induced by Compound 48/80. The data from the current study further confirmed that mast cell activation plays a central role in exacerbating IIR injury, although the mechanism governing mast cell activation during IIR is yet to be explored.

Uncontrollable inflammation and neutrophils sequestration in inflamed tissues also contribute to IIR injury, and selectins and intercellular adhesion molecules (ICAMs) by the activated endothelium induce neutrophils migration to injurious site [27]. Compton et al. have reported that tryptase released from mast cell degranulation plays a central role in attracting leukocytes infiltration and migration to ischemic tissues [28]. And treatment with mast cell stabilizer cromolyn sodium can reduce the expressions of ICAM-1 in the lungs in a rat model of pancreatitis-associated lung injury and downregulate IL-6 level [29]. These findings point to the importance of mast cell degranulation, through increased release of proinflammatory mediator, in inducing neutrophil migration to inflamed tissues. In the present study, we found that, during IIR, mast cell degranulation resulted in more neutrophils infiltration into small intestine evidenced as significant elevations in MPO activities, ICAM-1 protein expression, and IL-6 concentration; most importantly, cromolyn sodium abolished the alterations induced by IIR and prevented Compound 48/80-mediated exacerbation of IIR injury.

Previous study reported that sevoflurane preconditioning prevents from acute lung injury induced by aortic ischemia and reperfusion through reducing pulmonary neutrophil accumulation [10]. We also found that sevoflurane preconditioning significantly downregulated the MPO activities, ICAM-1 protein expression, and IL-6 concentrations, indicating that inhibiting neutrophil sequestration and the subsequent systemic inflammation may be the potential mechanism of attenuating IIR injury. It is well known that the delay in diagnosis and treatment of IIR injury contributes to the continued high in-hospital mortality rate [30]; the findings from the current study suggested that preventive administration of sevoflurane may be a promising approach in alleviating postoperative intestinal ischemia and mortality.

Sevoflurane is a preferable clinical choice in patients diagnosed to have mastocytosis, a group of rare disorders caused by the presence of too many mast cells and CD34+ mast cell precursors, without inducing mast cell degranulation [12]. It is well known that sevoflurane can mimic the effect of brief ischaemic episodes and protect from ischemia/reperfusion injury; Annecke and the groups have demonstrated that sevoflurane preconditioning significantly alleviates ischemia reperfusion injury in an ex vivo model of heart without alterations of histamine, and the findings suggested the benefits provided by sevoflurane preconditioning are not involved in mast cell [13]. Under the current study, we adjusted exposure of rats with 1 MAC sevoflurane for 1 h in three consequent days in the presence or absence of mast cell activator. The results from the current study showed that sevoflurane preconditioning greatly attenuated IIR injury demonstrated by reductions in injury scores and down-regulations of neutrophil infiltrations and inflammation; however, those protections are reversed by mast cell activator Compound 48/80, whereas mast cell stabilizer cromolyn sodium still confers protections challenged by Compound 48/80. The data from the present study indicated that the protective roles of sevoflurane preconditioning are independent of inhibiting mast cell.

Overproduction of reactive oxidative species (ROS) by NADPH oxidase is generally considered to play a critical role in the pathogenesis of ischemia reperfusion injury, and there are a number of NADPH homologs, such as Nox1, Nox2 (also named as gp91^phox^), and Nox3-4 [31]. It is should be noted that Nox2 is predominantly expressed within epithelial cells. Guan et al. demonstrated that intracellular NADPH concentration, in villus tip cells in intestine were significantly rapidly increased even after short-term ischemia [32]. Bedirli et al. recently reported that sevoflurane preconditioning dramatically attenuated the oxidative stress in a rat model of sepsis [33]; moreover, Yang et al. have demonstrated that sevoflurane preconditioning confers neuroprotection by increasing antioxidant enzymes [11]. In agreement with the previous results, we also found that 75 min small intestinal ischemia and 2 h reperfusion led to significant increases in MDA level and gp91^phox^ and p47^phox^ proteins expression, and resulted in significant decreases in SOD activities, furthermore, preconditioning of IIR-rats with sevoflurane significantly reduced the IIR mediated alterations, indicating that sevoflurane preconditioning confers protections against IIR injury possibly through reducing oxidative stress.

In addition to IgE/Fc*ε*, the classical signal pathway of mast cell activation and several distinct nonimmunological stimuli also contribute to mast cell activation [34], including trauma and physical stress. Yoshimaru et al. proved in vitro that upregulated ROS activates mast cell by NADPH oxidase [35], Collaco et al. have demonstrated that inhibiting ROS can dramatically stabilize mast cell in vitro [36]; we previously found significant increases of mast cell activation and concomitant elevations in oxidative stress in a rodent challenged by IIR [22]. The results indicate that there is linkage between mast cell activation and superoxide production. Our current study extended findings of previous studies [22] by showing that NADPH oxidase is overexpressed during IIR which contributed to increased oxidative stress and MC activation, leading to exacerbation of IIR injury, and pretreating of rats with mast cell stabilizer cromolyn sodium not only reduced MC activation, but also attenuated NADPH oxidase overexpression and reduced ROS production and consequently attenuated IIR injury and increased survival rate. Pretreatments of rats with sevoflurane displayed significant elevations in oxidative stress during IIR in the presence of mast cell activator Compound 48/80; the results are in line with the findings of Kaida et al. who reported that mast cell degranulation per se contributes to increased oxidative stress [37].

The limitation of the current study is that we did not show the direct relationships between the sevoflurane preconditioning and neutrophil migration in the development of IIR injury; several studies so far have confirmed that sevoflurane preconditioning can inhibit neutrophil infiltrate into ischemic area [10]; therein, the present study did not focus on the underlying mechanism by which sevoflurane preconditioning affects neutrophil sequestration.

In a summary, we have shown that mast cell activation, through increased release of mediators, contributes to deleterious injury induced by IIR and that mast cell stabilizer inhibits mast cell activation, attenuates IIR injury, and enhances postischemic mortality; sevoflurane preconditioning attenuates IIR injury possibly through blocking neutrophil sequestration and reducing oxidative stress but not through inhibiting mast cell, while the underlying mechanism merits further study.
